# Advanced Sulfur-Silicon Full Cell Architecture for Lithium Ion Batteries

**DOI:** 10.1038/s41598-017-17363-5

**Published:** 2017-12-08

**Authors:** Rachel Ye, Jeffrey Bell, Daisy Patino, Kazi Ahmed, Mihri Ozkan, Cengiz S. Ozkan

**Affiliations:** 1 Mechanical Engineering Department, University of California Riverside, 900 University Ave., Riverside, CA 92521 USA; 2 Material Science and Engineering Program, University of California Riverside, 900 University Ave., Riverside, CA 92521 USA; 3 Electrical and Computer Engineering Department, University of California Riverside, 900 University Ave., Riverside, CA 92521 USA

## Abstract

Lithium-ion batteries are crucial to the future of energy storage. However, the energy density of current lithium-ion batteries is insufficient for future applications. Sulfur cathodes and silicon anodes have garnered a lot of attention in the field due their high capacity potential. Although recent developments in sulfur and silicon electrodes show exciting results in half cell formats, neither electrode can act as a lithium source when put together into a full cell format. Current methods toward incorporating lithium in sulfur-silicon full cells involves prelithiating silicon or using lithium sulfide. These methods however, complicate material processing and creates safety hazards. Herein, we present a novel full cell battery architecture that bypasses the issues associated with current methods. This battery architecture gradually integrates controlled amounts of pure lithium into the system by allowing lithium the access to external circuit. A high specific energy density of 350 Wh/kg after 250 cycles at C/10 was achieved using this method. This work should pave the way for future researches into sulfur-silicon full cells.

## Introduction

Lithium-ion batteries (LiBs) outperform other battery technologies on the market, making them the choice for consumer electronics and electric vehicles (EVs). However, performance and cost demands have begun exceeding the capabilities of current LiB technology. Researchers have turned towards next generation battery materials to procure cheaper, higher capacity batteries^1–7^.

Current LiBs utilize a cathode made from lithiated metal oxides, such as lithium nickel manganese cobalt oxide (NMC). The cathode is traditionally countered by a graphite anode, although some in the industry have recently started incorporating silicon into the anode (1–5%). The advantages to this combination are high rate capabilities, low capacity degradation, and long lifetime. The disadvantages are a limited energy density, with NMC/Graphite having the highest theoretical energy density at 605 Wh/kg, and high cost of $180/kWh. To reduce costs, researchers have turned toward more energy dense and cheaper materials.

Sulfur is an attractive cathode material due to its theoretical capacity of 1675 mAh/g. However, implementation of sulfur has been slow due to its inherent problems including polysulfide shuttling, volumetric expansion, and poor conductivity^1,2,8–11^. Polysulfide shuttling results from higher order polysulfides dissolving in the electrolyte, causing long term capacity degradation and slowing reaction kinetics during runtime^12^. Volumetric expansion results from sulfur expanding (80%) during lithiation/delithiation which causes mechanical degradation to the electrode’s conductive network^12^. Finally, sulfur’s insulating properties affect the electrode’s rate capabilities. Fortunately, researchers have discovered methods to alleviate these issues ranging from mechanical barriers, to porous carbon networks, to other chemical methods^13–17^. Promising performance from these solutions have resulted in much fervor surrounding sulfur.

The current anode of choice is silicon for its high theoretical capacity of 4200 mAh/g. Silicon faces two challenges - poor conductivity, and volumetric expansion^18–21^. During lithiation/delithiation, silicon’s volume changes 400% which mechanically pulverizes the electrode, and degrades its cycle life and rate capabilities^21,22^. To alleviate these issues, researchers utilize novel methods including nano silicon structures, conductive additives, and binders^23–29^. Ultimately, the immense focus on solving each electrode’s issues has resulted in less research effort on combining a sulfur cathode and silicon anode in a full-cell configuration.

A full cell using sulfur and silicon electrodes is attractive for several reasons. Sulfur and silicon are environmentally benign and abundant. Furthermore, theoretical energy density of a sulfur silicon full-cell (SSFCs) is 1982 Wh/kg, far exceeding the theoretical energy density of current LiBs while only potentially costing $13/kWh. However, a major restriction for SSFCs is the lithium source. Currently, researchers utilize pre-lithiated materials such as lithium sulfide or lithium silicide, allowing for energy densities up to 600 Wh/kg. However,these full cells suffer from short cycles lives, typically less than 50 cycles, while the material used require specialized equipment and face restrictions in processing^30–32^.

Here, we present an advanced LiB architecture utilizing a sulfur cathode and silicon anode with lithium source integrated into the Si anode that can bypass these issues. The SSFC exhibits an energy density of 350 Wh/kg for 260 cycles at C/10. To the best of our knowledge, an SSFC with this architecture has not been reported.

## Results and Discussion

**Figure 1:**
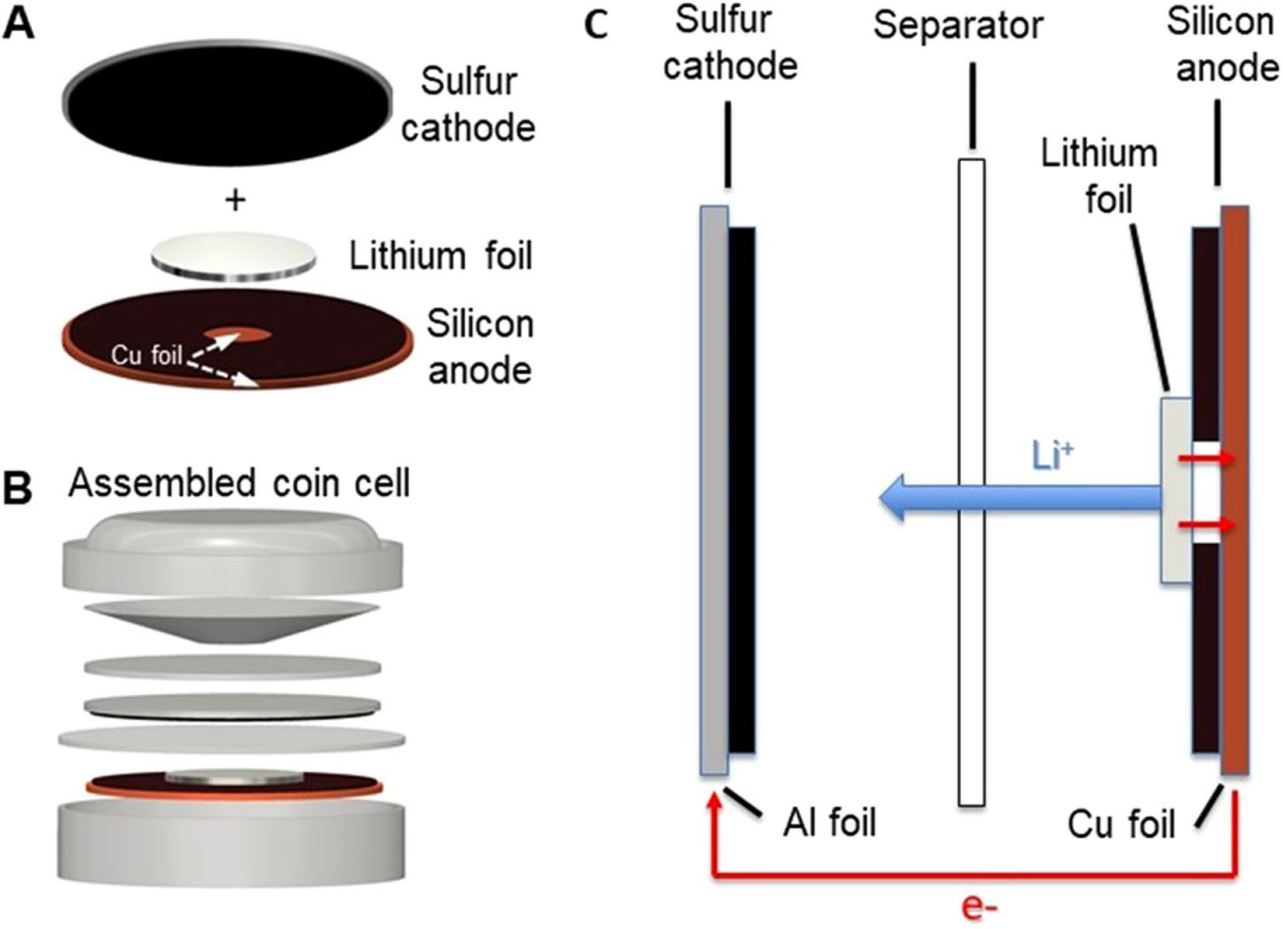
(**A**) SSFC battery architecture set up. (**B**) Assembled SSFC coin cell schematic. (**C**) SSFC Cross sectional discharge schematic.

Electrodes for SSFCs were constructed using a facile process. Shown in Fig. 1A, the silicon electrode is patterned to create an access point for the lithium chip, sitting on top of the silicon electrode, to contact the current collector. The access point allows electrons to transfer from lithium to positive terminal, Fig. 1C, creating a complete circuit. During discharge, the surface area of the lithium chip with direct access to the outer circuit alongside with the silicon anode should act as a lithium source. This provides lithium ions to the cathode through electrolyte while electrons travel to the cathode through the outer circuit. During charge, due to the reducing property of lithium, lithium ions will preferentially react with the silicon anode instead of attaching to the lithium chip. As cycling increases, lithium without direct access to the outer circuit also gains access to electrons through the silicon anode, and gradually integrates into the system. This results in an increase in capacity, discussed in the following sections. Each SSFC requires roughly 6.44 mg of lithium, accounting for the lithiation of sulfur and silicon along with consumption of lithium by the SEI. (See supplementary document for detailed calculation) To ensure enough lithium is available in the system, each cell is loaded with 8 mg of lithium.

**Figure 2:**
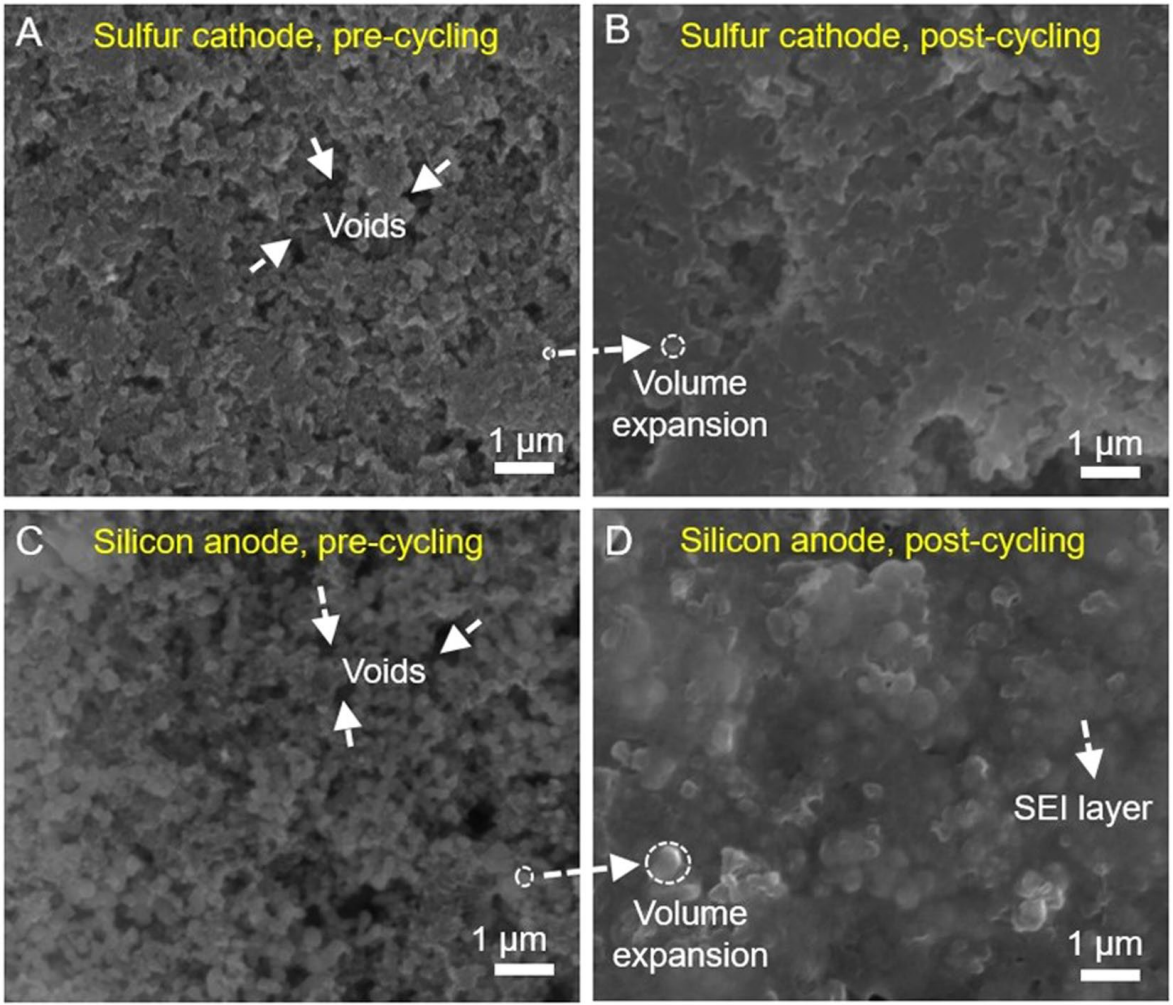
SEM images of sulfur cathode (**A**,**B**) and silicon anode (**C**,**D**) before and after 300 charge/discharge cycles, respectively.

The morphology of the electrodes was examined using SEM, shown in Fig. 2. Figure 2A and C show the surface of the sulfur and silicon electrodes respectively before they were cycled in the SSFC. Figure 2B and D show the post-cycling morphology of the corresponding electrodes. Pre-cycling SEM shows the electrode materials are loosely packed, with large void spaces existing after calendaring. Post-cycling SEM shows less void space due to the volumetric expansion of active materials and the formation of SEI products during lithiation^3,18^. This indicates that both of the electrodes have underwent lithiation and is a proof of silicon being utilized as the anode and sulfur as the cathode. The post-cycling SEM of the lithium foil after 310 cycles (Figure S2) was also done to further confirm that silicon is being used as the anode.

**Figure 3:**
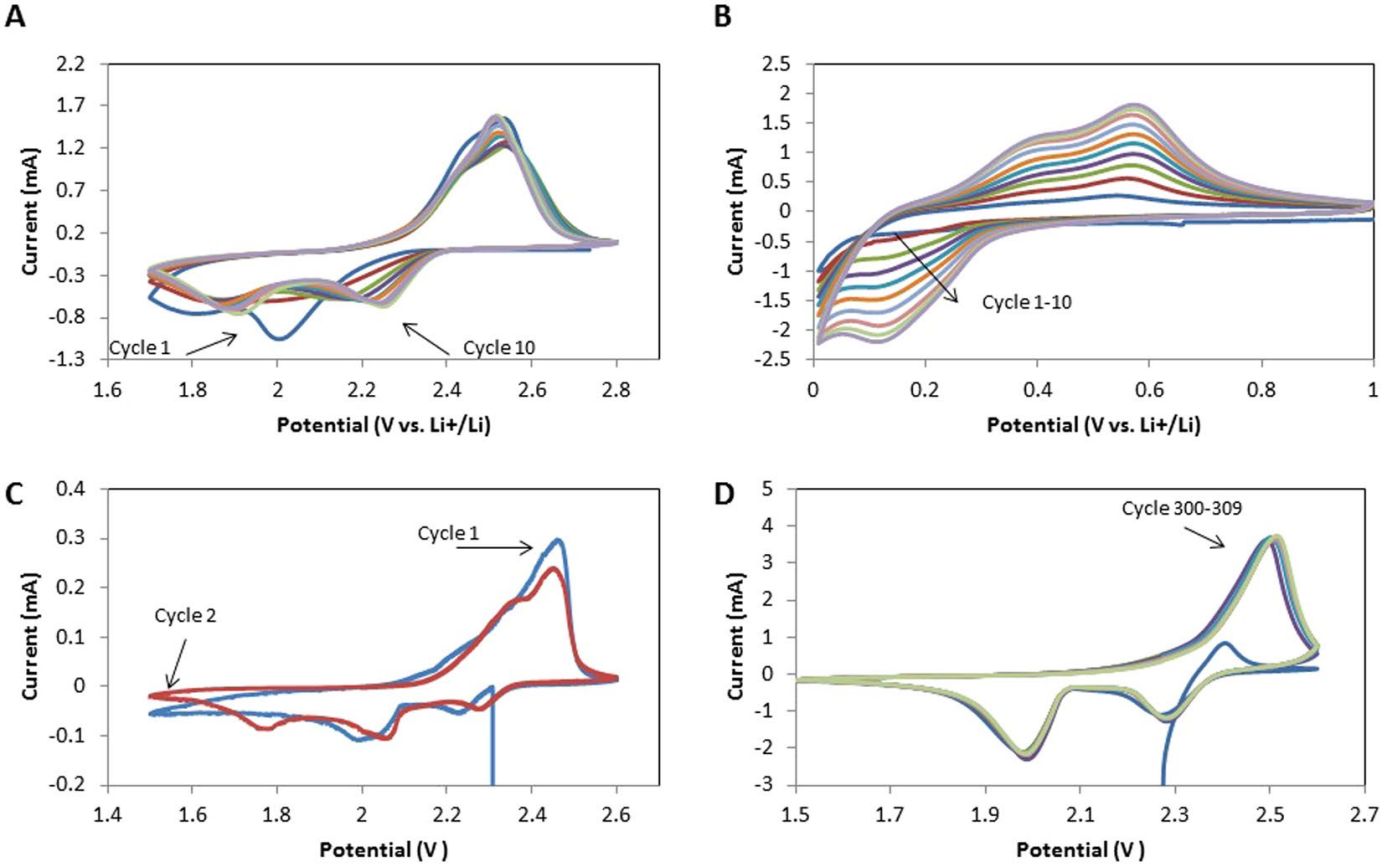
(**A**) Cycles 1–10 for the sulfur electrode at a scan rate of 0.1 mVs^−1^. (**B**) Cycles 1–10 for the silicon electrode at a scan rate of 0.1 mVs^−1^. (**C**) CV of Cycles 1–2 for the SSFC at a scan rate of 0.05 mVs^−1^. (**D**) CV of Cycles 300–309 for the SSFC at a scan rate of 0.1 mVs^−1^.

CV was performed at a scan rate of 0.1 mV s^−1^ over cycles 1–10 for both sulfur and silicon half-cells. SSFC CV was conducted at 0.05 mV s^−1^ and 0.1 mV s^−1^ respectively for cycles 1–2 and 300–309. The 0.05 mV s^−1^ scan rate was used to accommodate the aforementioned requirements for lithium integration during cycles 1–2. Figure 3A and B show CV profiles for cycles 1–10 of sulfur and silicon half-cells respectively. Shown in Fig. 3A, the sulfur half-cell exhibits typical characteristics of chemical reactions between sulfur and lithium ions with two cathodic peaks at 1.9 V and 2.25 V followed by an anodic peak at around 2.5 V^33,34^. The notable difference for cycles 1 and 2 is the offset peaks at 1.8 V and 2 V. Peaks shifting towards a higher potential indicates a higher ionic conductivity stemming from increased polysulfides and SEI formation^35^. Shown in Fig. 3B, the silicon half-cell shows typical cathodic peaks at 0.18 V and 0.1 V with anodic peaks at 0.4 V and 0.6 V. The cathodic and anodic peaks corresponding to lithiation/delithiation increase in intensity over time, resulting from lithiation of the native SiO_2_ layer and lithium gaining access to additional silicon^36^. The peak associated with SEI formation (0.67 V) does not exist after the first cycle, showing bulk SEI formation has been achieved^36^.

Figure 3C and D show CV profiles for the SSFC for cycles 1–2 and 300–309 respectively. Figure 3C and D exhibit a similar electrochemistry to Fig. 3A resulting from interactions between lithium ions and sulfur dominating the SSFC chemistry. In Fig. 3C, the first cycle has cathodic peaks at 2 V and 2.2 V resulting from limited amounts of lithium participating in the first discharge. Cycle two has an additional peak around 1.8 V, which we hypothesize to be a result of the negative voltage potential between the non-participating lithium and silicon. The resulting equilibrium voltage equals to the difference between the original potential of unlithiated sulfur and lithium (~2.8 V), and the potential between silicon and lithium (~1 V). The extra anodic peak at 2.35 V is also caused by non-participating lithium. This causes a negative potential between lithium and silicon (~0.15 V), shifting the normal peak at 2.5 V down to 2.35 V.

Figure 3D shows the CV profile for the SSFC once it has reached equilibrium. The two cathodic peaks at 2.0 V and 2.3 V followed by an anodic peak at 2.5 V match the electrochemistry of sulfur half-cell, Fig. 3A. This slight shift in peaks towards a higher potential represents complete activation of the lithium and further kinetic enhancement of the system^35^. The difference in current range between Fig. 3C and D is attributed to a change in the peak current, alluding to a higher capacity and reactivity^37^.

**Figure 4:**
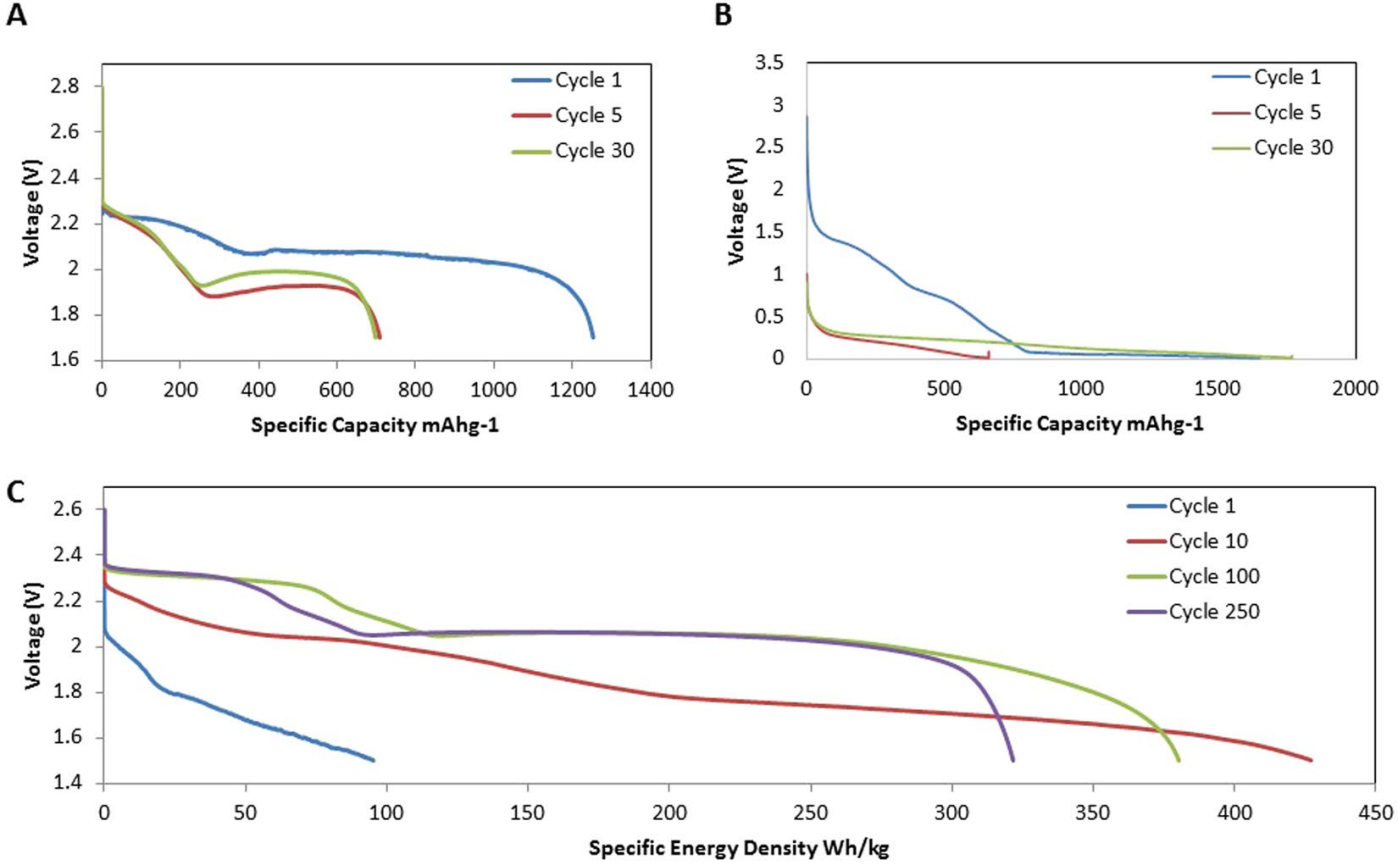
(**A**) Galvanostatic voltage profiles for the sulfur electrode at C/10 for selected cycles. (**B**) Galvanostatic voltage profiles for the silicon electrode at C/10 for selected cycles. C) Galvanostatic voltage profiles for the SSFC at C/10 for selected cycles.

The charge-discharge profiles for the SSFC, sulfur and silicon cells are shown in Fig. 4. The potential of the sulfur half-cell during its first discharge in Fig. 4A exhibits two long plateaus at 2.3 V and 2.1 V. The first long plateau at 2.3 V is associated with long chain polysulfide formation (Li_2_S_X_:x = 8,6,4)^38^. The second plateau at 2.1 V corresponds to the formation of Li_2_S_2_ and Li_2_S^38,39^. After the first cycle, the plateau at 2.1 V shifts to 1.9 V due to the enhanced kinetics, which concurs with the CV profile in Fig. 3A. The potential of the silicon half-cell during its first discharge in Fig. 4B exhibits a long plateau starting at 1.4 V. This corresponds to the formation of the solid electrolyte interphase^24,40^. The voltage plateau at 1.4 V disappears after the first cycle, and is replaced with a plateau at 0.2 V. This is in accordance with the cathodic peak seen in Fig. 3B. The CV and discharge profiles for the sulfur and silicon half-cells are also consistent with data reported in literature^3,29^.

Figure 4C shows the discharge profile for the SSFC. The first cycle has plateaus at 2 V and 1.8 V which concur with Fig. 3C. At cycle 2, an excess plateau at 1.8 V results from the equilibrium potential between sulfur, silicon, and non-participating lithium. This speculation is further proven by the change of the 1.8 V plateau, which becomes shorter as the test progresses, indicating less non-participating lithium. The voltage difference between the 10th and 100th cycles results from the cell conditioning and stabilizing by incorporating additional lithium over time. Once all the available lithium participates in the battery system, as shown in cycle 100 and 250, the voltage profile of the SSFC is in accordance with the sulfur half-cell. This proves that a stabilized SSFC act similar to a conventional full cell where cathode dominates the electrochemistry^30,31^.

**Figure 5:**
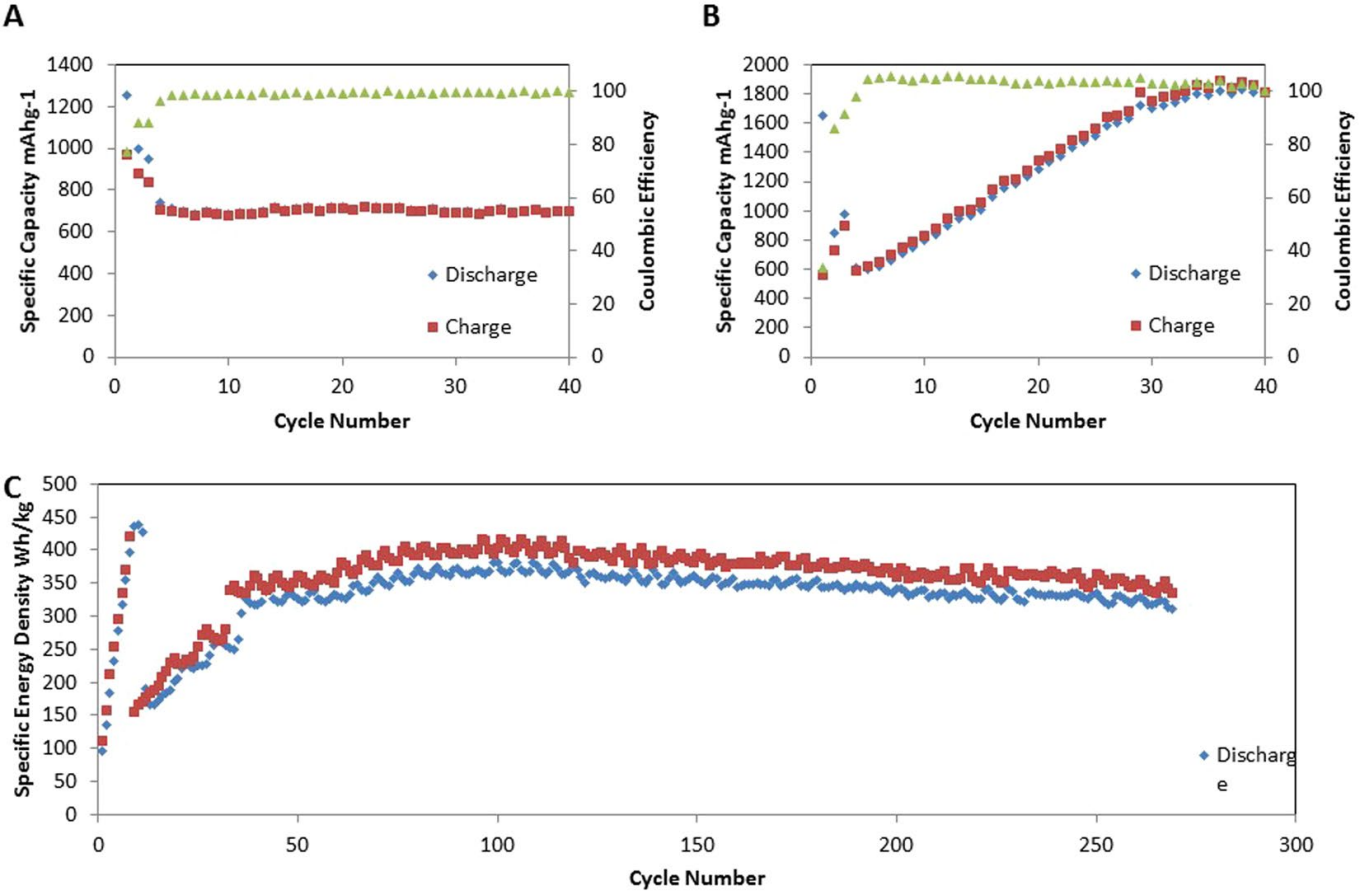
(**A**) Galvanostic cycling of the sulfur electrode at C/10 for 40 cycles. (**B**) Galvanostic cycling of the silicon electrode at C/10 for 40 cycles). Deep Galvanostic cycling of the SSFC at C/10 for more than 250 cycles.

Galvanostatic cycling of the sulfur and the silicon half-cell was carried out at a potential window of 1.7–2.8 V and 0.01–1 V respectively. In Fig. 5, the capacity for the batteries was measured at a rate of C/10 after being conditioned at C/50 for 3 cycles. The sudden decrease in performance at cycle 4 for both half cells is due to the rate change from C/50 to C/10. In Fig. 5A, the sulfur half-cell has an initial capacity of 1254 mAhg^−1^ and maintains a capacity of 700 mAhg^−1^ for 40 cycles with a coulombic efficiency greater than 99%. The decrease in capacity is attributed to SEI for mation, polysulfide shuttling, as well as mechanical degradation of the electrode. In Fig. 5B, the silicon half-cell has an initial capacity of 600 mAhg^−1^, and stabilizes at 1800 mAhg^−1^ within 40 cycles with a coulombic efficiency greater than 99%. The increase in capacity is attributed to the calendared electrode limiting the expansion of lithiated silicon and electrolyte penetration^41^. This coincides with Fig. 3B, wherein the overall CV curve of the silicon half-cell increases in intensity over time, alluding to a higher capacity^37^.

**Figure 7:**
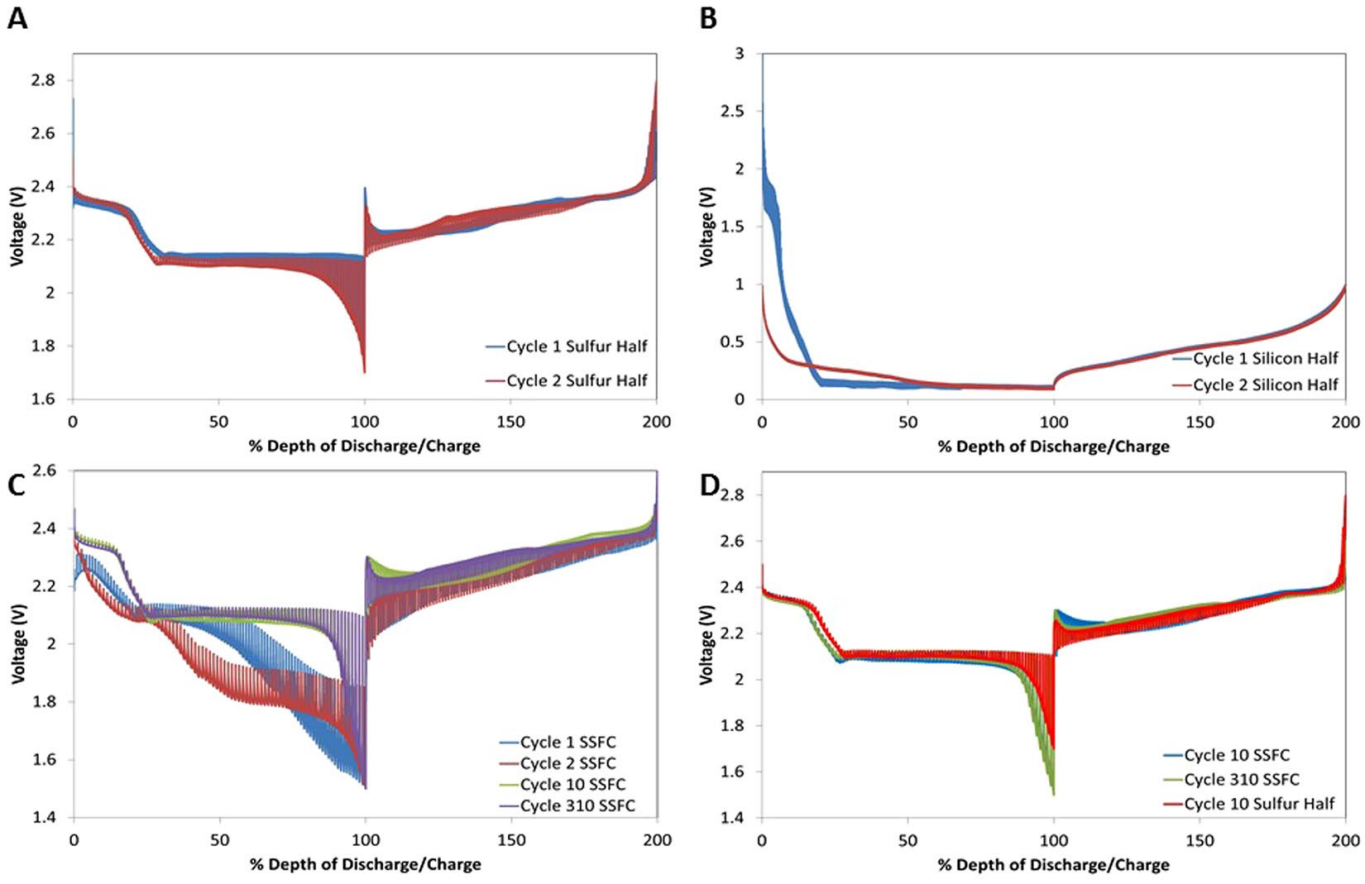
(**A**) GITT analysis on the sulfur electrode at C/50 with 10 minutes rest for cycles 1–2. (**B**) GITT analysis on the silicon electrode at C/50 with 10 minutes rest for cycles 1–2. (**C**) GITT analysis on the SSFC at C/50 with 10 minutes rest for cycles 1, 2, 10, 310. (**D**) GITT analysis comparing sulfur electrode at cycle 10 vs SSFC at cycles 10,310.

Figure 5C shows galvanostatic cycling for the SSFC. The energy density of the SSFC, which is calculated based on the total anode and cathode weight (see supplementary information for details), is recorded for 250 cycles. The wave like fluctuations in capacity results from temperature changes occurring inside the testing room. The initial energy density of the SSFC is 100 Wh/kg at C/50 then increases to 414 Wh/kg over 10 cycles. The sudden drop in capacity at cycle 11 is due to the current rate change from C/50 to C/10. The increase in energy density is attributed to the continuous integration of non-participating lithium, shown in Fig. 5C; this hypothesis is confirmed by Figs 3, 4, and 7. The SSFC has an energy density of 350 Wh/kg for over 250 cycles and a coulombic efficiency of approximately 95%. The fluctuation in coulombic efficiency from cycle 1 to 150 is due to the process of lithium integration, which creates a unique chemical reaction to the SSFC. When charging a conventional full-cell, lithium ions from the cathode travels to the anode while electrons travels through the outer circuit from cathode to anode as well, as a result, the anode materials are lithiated. Li-ions and the electrons are then returned to the cathode during discharge. In the SSFC, lithium starts on the anode side, thus discharge happens during the first cycle. We propose that during discharge, lithium ions from the lithium chip travel to the cathode through electrolyte, while the electrons from the lithium chip travels through its contact point with the current collector and joins the electrochemistry process. However, in later cycles, lithium that is not directly in contact with the current collector can only join the system by either transferring electrons through the relatively insulating silicon slurry or by lithiating the silicon slurry during charge. This additional lithiation increases the charge capacity, which in turns decreases the coulombic efficiency. Hence, the coulombic efficiency of cycles 1 to 150 are low and unstable despite the cathode operates with a stable coulombic efficiency of 99%, shown in Fig. 5A. After 150 cycles all required lithium is incorporated into the SSFC system and is actively participating in the redox reaction, however, excess lithium remains. During charge, lithium ions from the cathode plate onto the excess lithium chip while in parallel, lithium ions from the chip react with the silicon anode. As a result, the coulombic efficiency after 150 cycles have improved but are still in the range of 95% instead of being similar to the sulfur half-cell.

**Figure 6:**
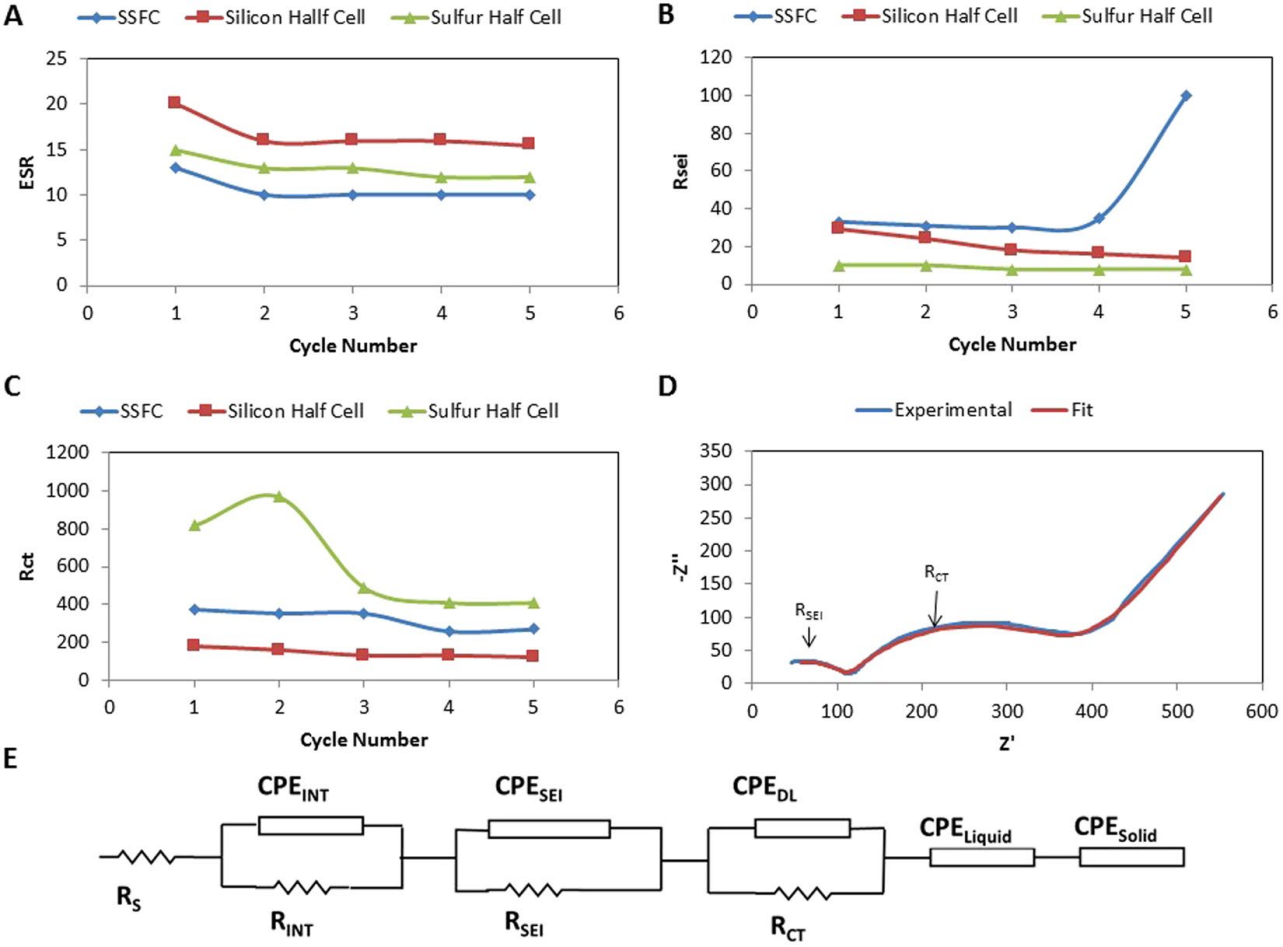
Impedance parameters during initial cycles for SSFC, silicon half-cell, and sulfur half-cell. (**A**) ESR. (**B**) R_CT_. (**C**) R_SEI_. (**D**) Experimental data SSFC cycle. 5 (**E**) EEC used to obtain parameters.

Electrochemical impedance spectroscopy, shown in Fig. 6, is a non-destructive method allowing us to investigate the integrity of electrode-electrolyte interface, passivation layers, electronic conductivity of electrode material, diffusion of lithium within electrode, and diffusion of lithium ions in electrolyte near electrode surface. Potentiostatic EIS is utilized to characterize the cells’ complex impedance by measuring the current response to a small sinusoidal voltage signal. Impedance is obtained for a selected number of frequency points between the bounds of 10 kHz and 10 mHz.

Figure 6E shows the electrical equivalent circuit used to model the impedance of lithium-ion cells at a fully charged state. A fit between the impedance response of the circuit and that of the cell is obtained by tuning the circuit parameter values. The constant phase elements (CPE) present in the circuit are capacitances that are spatially non-uniform. Equation 1 gives the formula used to calculate the impedance of CPE. Here Q is analogue to capacitance, and n is an ideality factor that is constrained between 0 and 1, while an ideality factor of 1 is identical to an ideal capacitor.

1 $${Z}_{CPE}=\frac{1}{Q{(j\omega )}^{n}}$$

In Fig. 6E, the value of equivalent series resistance (ESR) represents electrolyte conductivity. R_INT_ quantizes electronic conductivity within electrode matrix, while CPE_INT_ is a measure of the non-ideal capacitance that arises due to this finite conductivity. CPE_FILM_ and R_FILM_ quantize non-ideal capacitance and resistance associated with the passivating layers. CPE_DL_ measures the nature of the Helmholtz double-layer formed about the electrode-electrolyte interface, while R_CT_ determines the exchange current density. R_CT_ is an indicator of how facile electron exchange kinetics are at the interface. CPE_LIQUID_ quantizes diffusion of lithium ions in electrolyte near electrode surface. This diffusion impedance originates from the concentration gradient of lithium ions existing between the diffuse layer of charge and bulk electrolyte. CPE_SOLID_ represents solid state diffusion of lithium atoms within the electrode material after lithiation and before delithiation.

Figure 6A shows the evolution of ESR during initial cycling in the SSFC and the sulfur/silicon half-cells. ESR in all three cells show a stabilizing trend, which provides evidence of electrochemical durability. It is observed that the two half-cells show a larger ESR than the SSFC. A previous study has shown that electrolyte decomposition is worse in half-cells due to the presence of lithium-metal counter electrodes^42^. Figure 6C shows the change in R_CT_ during the initial cycles in the same cells. It provides evidence for sulfur having slower kinetics than silicon. All three cells show a stabilizing trend over the initial cycles.

Figure 6B shows how R_SEI_ changes for the three cells within the same cycling window. Here we observe that the SSFC has the highest resistance value when compared to silicon and sulfur half-cells. We propose that the method we utilized to lithiate the full-cell assembly contributed to this observation. Lithium metal placed within the SSFC formed its own SEI during the initial cycling while the lithium content was slowly integrated into the anode. While the chip lost its lithium content to silicon anode, the SEI layer formed on top of it remained. Additionally, another SEI layer formed on the silicon anode as it participated in active lithiation/delithiation reactions. Thus, SSFC exhibits SEI impedance that originate from the silicon anode, from conductive carbon added in sulfur cathode, and from the lithium metal itself used to lithiate the full-cell. We also observe a spike in R_SEI_ at the end of the 5th cycle. We hypothesize that this spike occurs due to the majority of SEI formation taking place on the silicon anode. We also observed that sulfur half-cell showed the lowest R_SEI_ value among the three cells. This is so because sulfur does not natively form any permanent passivation film similar to SEI layers observed in silicon or carbon electrodes. SEI impedance observed in our sulfur electrodes originate from the carbon additive added to the electrode matrix as conductive agent.

GITT, shown in Fig. 7, was employed to investigate changes in lithium diffusivity within the individual battery systems^43,44^. The batteries were subjected to current pulse intervals with a rate of C/50 for 10 minutes, followed by 10-minute rests until complete discharge/charge. In Fig. 7, the varying thickness of the voltage profiles represent varying lithium diffusivities in the system. Thinner voltage profiles indicate improved diffusivity while thicker voltage profiles represent the inverse^45,46^.

In Fig. 7A, the profile for the sulfur half-cell displays a slight decrease in voltage plateaus from cycles 1 to 2. This occurrence is also observed in Figs 3 A and 4A, and is attributed to the change in ionic and electric conductivity caused by the incremental SEI formation and polysulfide shuttling^44^. As seen in Fig. 7B, the silicon half-cell experiences a voltage shift within the first two cycles; this is attributed to SEI formation, coinciding with Fig. 4B. However, voltage profiles and diffusivity equilibrate by the second cycle, indicating that the silicon half-cell has faster kinetics than the sulfur half-cell as inferred by Fig. 6C. Hence, it is determined that the kinetics of sulfur half-cell is the limiting factor for the diffusivity of the SSFC.

Figure 7C shows the GITT profile for the SSFC. Figure 7C depicts the voltage profiles of the SSFC resembling the sulfur half-cell, revealing plateaus at 2.3 V and 2.1 V after reaching equilibrium. However, the first cycle of the SSFC shows a discharge profile offset from the sulfur half-cell; this is attributed to limited lithium participation in the first cycle. The excess voltage plateau in cycle 2, at roughly starting at 50% depth of discharge, alludes to the Li incorporation issues associated with the architecture of the cell. The broad voltage fluctuation in cycles 1–2’s GITT profile indicate a nonuniform material utilization caused by part of the electrode not being lithiated with the limited participating lithium, supporting the aforementioned speculation. At cycle 10, the voltage profile of SSFC already resembles a sulfur half-cell. The observable change in diffusion in cycle 2 to 10 is a result of total lithium utilization allowable in the system. This change in the voltage profile comparing to normal cycling, Fig. 4C, is due to the pulsed discharge currents of GITT progressing the cell at a faster rate allowing complete lithium integration by cycle 10. Once reaching complete lithium utilization, the diffusivity of the system continues to improve from cycles 10 to 310. Thinner voltage profiles as well as a higher voltage plateau are observed in the subsequent cycles, which is a result of enhanced kinetics.

Figure 7D compares the diffusivity of SSFC to the sulfur half-cell, wherein we see a notable difference within the early cycles. At 80–100% depth of discharge, the observable difference in diffusivity from the half-cell to SSFC is caused by the charge transfer resistance of the silicon anode. Similarly, once the cell starts to charge, the notable difference in diffusivity profiles at 0–20% depth of charge is a result of charge transfer resistance in the cathode for the SSFC. Ultimately, Fig. 7D depicts the SSFC voltage profile continues to coincide with that of the half-cell once it has developed a complete utilization of lithium.

## Conclusion

Herein, we have presented a simple alternative to prelithiated sulfur-silicon full cell systems by allowing lithium the access to external circuit. In addition to bypassing the complications of prelithiated cells, this method allows for the controlled loading of lithium to compensate for SEI formation and lithium degradation, prolonging the cycle life of the full cell. As a new full cell configuration for next generation lithium ion batteries, the SSFC demonstrates an energy density of 350 Wh/kg over 250 cycles at C/10. Furthermore, this is the first time, to the best of our knowledge, a sulfur silicon full cell has been fully characterized using EIS, CV and GITT. The results presented will pave the way for new research into sulfur and silicon full cells.

## Experimental Details

### Material Synthesis

The SSFCs consist of a sulfur cathode and a silicon anode. The sulfur cathode was made with 20 wt% Poly(acrylic acid) (PAA, 1800 g/mol,Sigma-Aldrich) and 80% wt% acetylene black sulfur composite(ABS). The aforementioned ABS was made by dissolving 200 mg of Sulfur (S, 99.998% trace metals basis, Sigma-Aldrich) in 20 ml of Dimethyl Sulfoxide (DMSO, Fisher Chemical) at 90 C, heated by a heating jacket (Brisk Heat). 129 mg of Acetylene black (Alfa aesar, 50% compressed) was then added to the solution, the solution was stirred for 3 hours before the heating jacket was removed, and the solution was allowed to cool while stirring. The resulting ABS composite was then washed by anhydrous ethanol (Decon Labs, Inc.) several times to ensure the removal of DMSO and dried at 60 C for 24 hours. To make the sulfur electrode, Poly (acrylic acid) (Sigma Aldrich, 450,000) and ABS were mixed with 1-Methyl-2-pyrrolidinone (NMP, Sigma-Aldrich) and then casted on a large piece of aluminum chip (Alfa Aesar, 0.025 mm thickness, 99.45% purity) by a doctor blade (MTI Automatic Thick Film Coater, BYK Doctor Blade). The casted electrode sheet was then dried in a convection oven (Cole-Parmer, Stable Temp) at 60 C for 24 hours. The silicon electrode was made with 40 wt% of commercial silicon (GNM Silicon nanoparticles 80 nm), 25 wt% Acetylene black (Alfa aesar, 50% compressed), and 35 wt% Poly (acrylic acid) (Sigma Aldrich, 450,000). The materials were mixed and sonicated in ethanol and then casted on a large copper chip (Alfa Aesar, 0.025 mm thickness, 99.8% purity) with a doctor blade (BYK) and was then dried at 60 C for 24 hours. Both electrodes were calendared with a 0.04 mm calendar gap using a calendaring machine (IRM) before being constructed into a coin cell.

### Physical Characterization

The morphology of the electrode pre and post cycling was observed by scanning electron microscopy (NovaNanoSEM 450).

### Electrochemical Characterization

To make the SSFC battery, a silicon electrode (16 mm in diameter) was first put inside a negative cap (MTI type 2032 coin cell case) and a piece of lithium (MTI Lithium Chip 15.6 Dia × 0.25t mm) with corresponding weight (4–6 mg depending on electrode weight, with adjustments for SEI consumption) was adhered to the top of the silicon electrode inside an Ar filled glovebox (H_2_O < 0.5 ppm, O_2_ < 0.2 ppm, Vacuum Atmosphere Co.) to form a complete circuit. The amount of lithium needed was calculated based on the electrode weights and SEI lithium consumption of the half-cells. Next, separators (Celgard 25um 3501) of various sizes were placed on top to prevent any possibility of shorting. Sulfur electrode (16 mm in diameter) was then placed on top followed by two spacers, a spring, and the positive cap were added with the electrolyte in between (1:1 DOL:DME, 1 wt% LiNO_3,_ 1 M LiTFSI). The battery was then sealed using a battery crimper (MTI, MSK-160D). The battery was tested under room temperature with a Bio Logic (BCS 810 Testing Module) using different testing methods, including Galvanostatic Cycling with Potential Limitation (GCPL), Cyclic Voltammetry (CV), Potentiostatic Electrochemical Impedance Spectroscopy (PEIS) and Galvanostatic Intermittent Titration Technique (GITT) in voltage window ranging from 1.5 V to 2.6 V. The same tests were also performed for the sulfur half-cell (between 1.7 V to 2.8 V) and the silicon half-cell (between 0.01 V to 1 V). The Sulfur weight percentage in the Acetylene Black Sulfur composite (ABS) was measured using thermogravimetric analysis (TGA), showing 57% weight sulfur. The SSFC and sulfur half-cell were conditioned with a current rate of 0.175 mA (C/50), and cycled at 0.875 mA (C/10). The silicon half-cell was conditioned at a current rate of 0.336 mA (C/50), and cycled at 1.68 mA (C/10).

### Data Availability

The authors declare that [the/all other] data supporting the findings of this study are available within the paper [and its supplementary information files].

## Electronic supplementary material

Supplementary Information
