# Supplementary material for: Advanced Sulfur-Silicon Full Cell Architecture for Lithium Ion Batteries
Source: Sci Rep. 2017 Dec 8;7:17264. doi: 10.1038/s41598-017-17363-5 (PMC5722921; doi:10.1038/s41598-017-17363-5)
Supplement: Supplementary file 1 — Supplementary Information [file 41598_2017_17363_MOESM1_ESM.doc]

supporting information for

Subject areas

Lithium-ion Batteries, Silicon, Anode, Sulfur, Cathode

Correspondence and requests for materials should be addressed to

C.S.O. ([cozkan@engr.ucr.edu](mailto:cozkan@engr.ucr.edu)) and M.O. ([mihri@ece.ucr.edu](mailto:mihri@ece.ucr.edu))

**Advanced Sulfur-Silicon Full Cell Architecture for Lithium Ion Batteries**

**Rachel Ye^1§^, Jeffrey Bell^2§^, Daisy Patino^2^, Kazi Ahmed^3^, Mihri Ozkan^3*^, and**

**Cengiz S. Ozkan^1,2*^**

^1^Mechanical Engineering Department, University of California Riverside, 900 University Ave. Riverside, CA 92521

^2^Materials Science and Engineering Program, University of California Riverside, 900 University Ave. Riverside, CA 92521

^3^Electrical and Computer Engineering Department, University of California Riverside, 900 University Ave. Riverside, CA 92521

**^§^** = (Designates equal contributions)

**Cost calculation**

According to data found online the cost of energy by producing NMC batteries is 180$/kWh while the energy density of said battery is at 1/3 of its theoretical energy density. This means that the cost will be $60/kWh if NMC/graphite battery is utilized at its specific energy density. To get an estimation of the cost to produce such energy by using SSFC cells, we have gathered the cost of raw materials from sigma Aldrich, as listed below, to estimate the cost difference between the material of NMC/graphite full cell and SSFC.

| Material | NMC | Graphite | Sulfur | Silicon | Lithium |
| --- | --- | --- | --- | --- | --- |
| Cost($/g) | 15.1 | 0.50 | 3.21 | 0.77 | 2.1 |

For an NMC/graphite full cell, every 1g of NMC will need 0.51g of graphite according to the weight balance equation of a full cell battery, with the NPC capacity being 170mAh/g, and the graphite capacity being 330mAh/g:


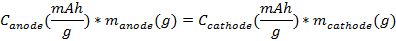


As such, the overall cost of a NMC/graphite battery will be


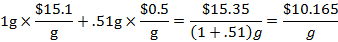


While the weight balance situation of a SSFC is calculated to be 1g of Li_22_Si_5_ weight balances with 1.174g of S. 1g of Li_7_Si_2_ using equation x. Since Li_22_Si_5_ consists of 52.1 wt% Li and 47.9 wt% of Si according to its molecular mass, a balanced SSFC will need .521g of Li, .479g of Si, and 1.174g of S. And such the overall cost of a SSFC battery will be


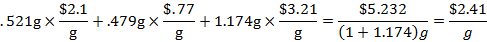


Thus, according to the calculated overall price, the overall cost ratio of a NMC/graphite full cell to a SSFC is $10.165/$2.41 = 4.22:1

With the SSFC having an theoretical energy density of 1968 kWh/kg, which is 3.25 times of the NMC/graphite full cell, the lowest cost needed to produce a SSFC can be


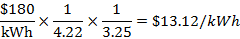


**Rates calculation**

The rates for the half-cells are calculated based on the following equation:


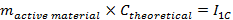


Where C is the theoretical capacity of such active material, and the 1C rate is defined as the battery will need 1 hour to charge and discharge if it is at its theoretical capacity.

As such, the rate for sulfur half-cell was calculated to be:


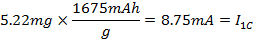


Which also allow us to calculate the rates C/50 and C/10.


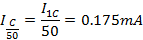


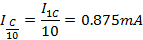


Using the same method, the rate for silicon half-cell was also calculated:


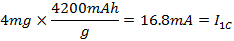


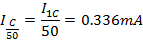


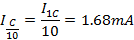


Since the same electrodes were used for full cell assembly, and the electrodes weren’t properly weight balanced, the rate used for full cell cycling was the sulfur half-cell rate due to the sulfur electrode being the limiting factor.

**Specific Energy calculation**

The specific energy of the battery (E) is calculated based on the total capacity of the battery (Q), nominal voltage window of the battery (V), anode active material weight (m_A_) and cathode active material weight (m_c_), as shown in the equation below. (Note that although the battery’s anode and cathode material are not energy balanced, the actual mass is used for calculation instead of energy balanced weight)


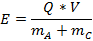


Using the first cycle as an example, the capacity is 0.4521 mAh. The specific energy is then calculated by:


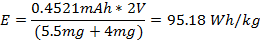


**Weight balance calculation**

To balance the anode and cathode in a full cell, the total capacity of the electrode should match with each other. To find the correct weight balance ratio of sulfur to silicon loading, the following calculation was carried out:


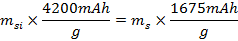


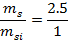


**Li amount calculation**

To decide the amount of lithium used to assemble the cell, the minimum amount of lithium needed by the system was first calculated. To do so, the lithium needed to lithiate the electrodes and the lithium consumed by the SEI during the condition cycles were added to get an estimation.

The Li needed to lithiate the electrodes can be calculated through molar mass, and should be decided by the higher lithium consumption electrode since the anode and cathode weren’t weight balanced.

For Sulfur:


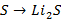


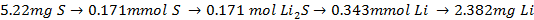


For Silicon


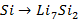


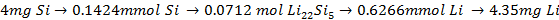


The SEI Li consumption of the conditioning cycles is estimated base on the Coulombic Efficiency of the half-cells, as shown:


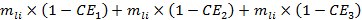


Where m_li_ is the Li used for that cycle of discharge, Since there is unlimited Li in half-cells, we can estimate m_li_ to be constant and equal to the theoretical Li mass that is calculated previously.

As such, the SEI Li consumption of the sulfur electrode was estimated to be:


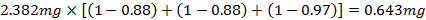


And the SEI Li consumption of the silicon electrode will be:


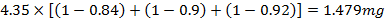


With these calculated, the minimum amount of Li we need to put into the system will be:


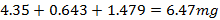


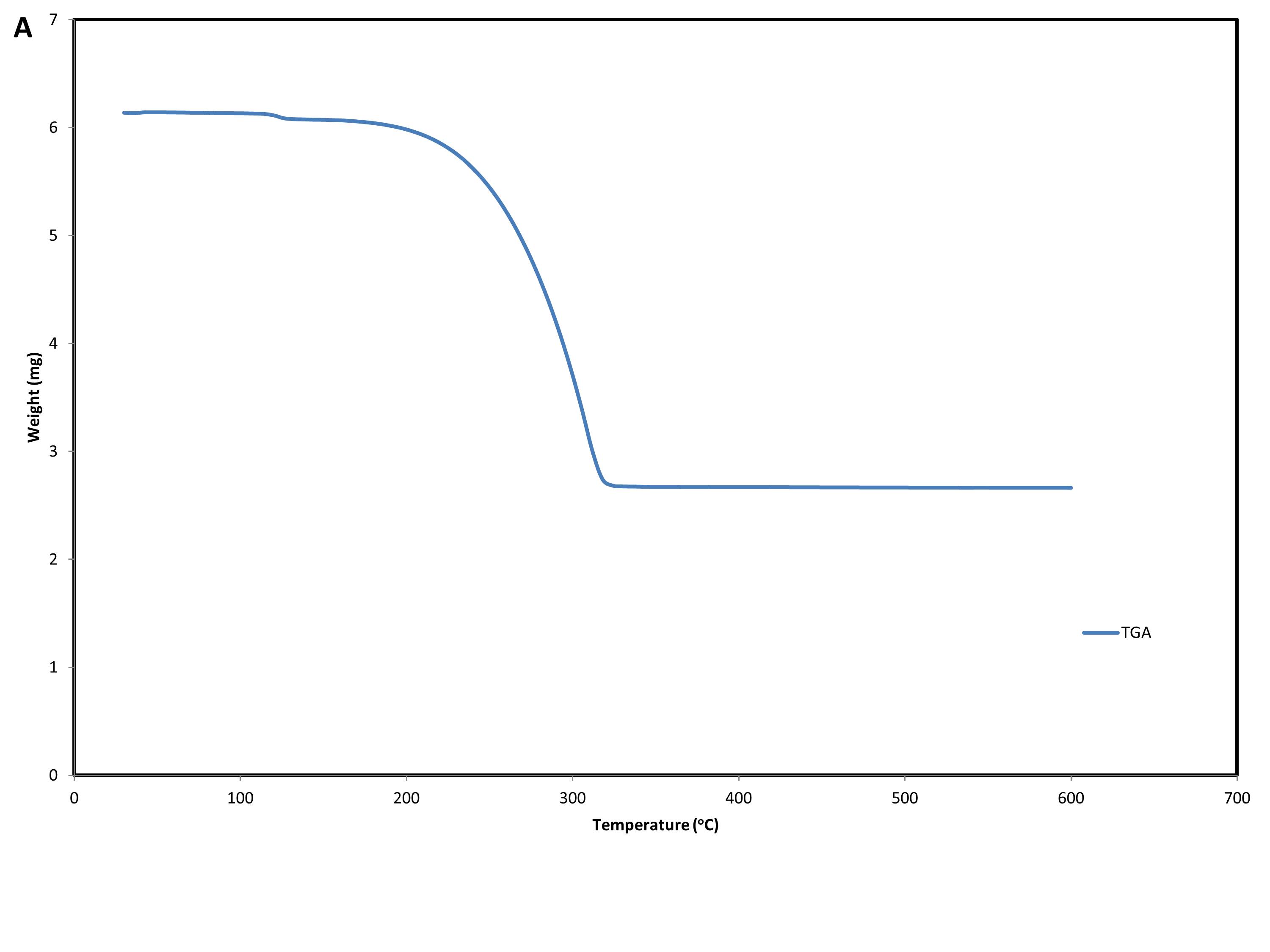


**Figure S1:** Thermogravimetric analysis of acetylene black sulfur composite.


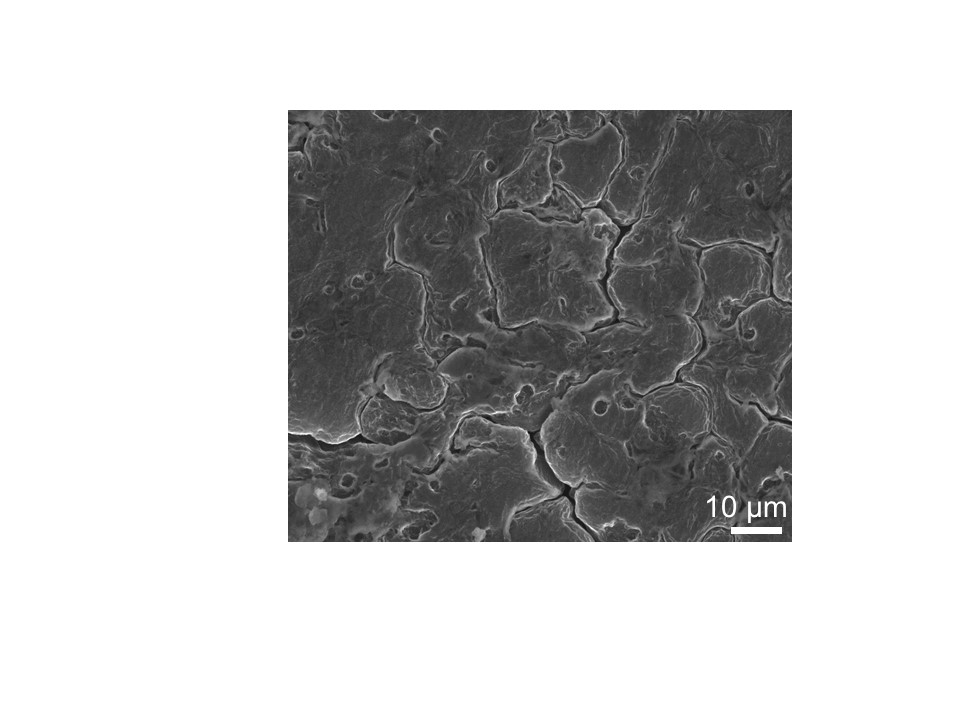


**Figure S2:** Post-cycling SEM of lithium foil inside of SSFC

Figure S2 shows the post-cycling SEM of the lithium foil after 310 cycles. Since the SSFC is not utilizing any dendrite suppression technique, dendrites should start to form on the foil as early as 20 cycles if the lithium was being used as the anode. The lack of dendrite formation in Figure 2E suggested that the foil was only used as the source of lithium rather than the anode.
